# The Enhancer–Promoter-Mediated *Wnt8a* Transcription During Neurite Regrowth of Injured Cortical Neurons

**DOI:** 10.3390/cells14050319

**Published:** 2025-02-20

**Authors:** Shr-Han Weng, Wen-Ling Liao, Linyi Chen

**Affiliations:** 1 Institute of Molecular Medicine, National Tsing Hua University, No. 101, Section 2, Kuang-Fu Road, Hsinchu 300044, Taiwan; shan960517@gmail.com (S.-H.W.); zoe880619@gmail.com (W.-L.L.); 2 Department of Medical Science, National Tsing Hua University, No. 101, Section 2, Kuang-Fu Road, Hsinchu 300044, Taiwan

**Keywords:** *Wnt8a*, neurite regrowth, injured cortical neurons, brain injury, epigenetic regulation

## Abstract

Brain injuries can result from accidents, warfare, sports injuries, or brain diseases. Identifying regeneration-associated genes (RAGs) during epigenome remodeling upon brain injury could have a significant impact on reducing neuronal death and subsequent neurodegeneration for patients with brain injury. We previously identified several WNT genes as RAGs involved in the neurite regrowth of injured cortical neurons. Among them, the expression of the *Wnt8a* gene increased most significantly during neurite regrowth, indicating its potential to promote neuronal regeneration. In this study, we investigated the regulatory mechanism of *Wnt8a* transcription. An algorithm was developed to predict the novel enhancer regions of candidate genes. By combining active enhancer marks, histone H3 lysine 27 acetylation (H3K27ac), and histone H3 lysine 4 mono-methylation (H3K4me1), we identified a candidate enhancer region for *Wnt8a* located 1.7 Mb upstream and 0.1 Mb downstream of the *Wnt8a* gene. This region was organized into enhancers (Ens) 1–15. Enhancer RNA expression from the predicted En1–15 regions, DNA topological dynamics, and the activity of predicted enhancers were analyzed to validate the candidate active enhancers. Our findings showed that the En8, 9, 10, 14, and 15 regions expressed higher eRNAs during neurite regrowth. Notably, the En8-2 and En14-2 subregions showed significantly up-regulated H3K4me1 modification during neurite regrowth. Using chromatin conformation capture assays and enhancer–reporter assays, we delineated that the molecular regulation of *Wnt8a* transcription during neurite regrowth occurs through looped En8-promoter interplay.

## 1. Introduction

Brain injuries can result from accidents, warfare, sports injuries, strokes, or brain tumors. They can cause a wide range of symptoms and deficits, depending on the affected brain regions. The failure of neural repair following a brain injury can lead to permanent disabilities. The regeneration of injured brain neurons is often limited by gliosis, inflammation proximal to the injury sites, and reduced transcription for neuronal growth in adult brains. Although brain neurons possess intrinsic regeneration potential, it is often neglected in tissue and brain models. Thus, this study aims to identify regeneration-associated genes (RAGs) in injured cortical neurons during neurite regrowth.

Transcription in the eukaryotic cells occurs in the nucleus, where DNA is typically wrapped into nucleosomes and higher-order chromatin structures. Therefore, mechanisms that reduce histone–chromatin interactions enable binding between DNA, transcription factors, and RNA polymerases, leading to successful gene expression. Post-translational modifications, including the acetylation, phosphorylation, ubiquitination, methylation, sumoylation, and ADP ribosylation of proteins, are major regulatory mechanisms that govern the activity of target proteins, protein–protein interactions, intracellular distribution, and, thus, biological functions [1]. To accommodate transcriptional regulators within the limited space of the nucleus, genomes are organized at multiple levels. Chromatin organization is divided by interactions between functionally distinct compartments, each domain referred to as topologically associating domains (TADs). Interaction among TADs, often derived from Hi-C results [2,3], may drive the expression of genes. In vertebrates, cohesin-mediated CTCF loops between TAD boundaries and RNA polymerase-anchored chromatin loops can separate the promoter and enhancer which only interact as needed [4].

Histone modifications thus play significant roles in diverse functional outcomes, e.g., mono-methylation of histone H3 at lysine 4 (H3K4me1) by the proteins Mll3 and Mll4 is regarded as a unique feature of enhancers due to the enhanced association of the chromatin remodeling complex BAF with enhancers [5]. Whereas, tri-methylation of histone H3 at lysine 4 (H3K4me3) is predominantly located within the promoters of RNA splicing regions, affecting transcription initiation and elongation rates through direct physical interactions with the pre-initiation complex [6,7,8]. Genomic enhancers serve in part to modulate transcription when interacting with promoters. These enhancers likely contain transcription factor binding sites, attract co-activators and co-repressors [9], and participate in DNA looping [10,11,12] to interact with other enhancers and promoters in a specific cellular context. Thus, their activity has been implicated in normal physiology and diseases. Mammalian cells contain thousands of active enhancers, and about 1 million active enhancers are found in all human cells [13]. Enhancer RNAs (eRNAs), which act as markers for active enhancers, are noncoding elements synthesized by RNA polymerase II to modulate transcription through a variety of mechanisms, such as recruiting transcription factors and coregulators to specific enhancers, enhancing acetylation of H3 lysine 27 (H3K27ac), and directing chromatin accessibility for interaction with RNA polymerase II [14,15,16,17]. The presence of eRNAs has been shown to precede activation of nearby genes, and eRNAs may play a role in enhancing or stabilizing long-range interaction between promoter and enhancer, which is necessary for gene activation. At the genome scale, the level of eRNA transcription and the mRNA synthesis at nearby genes are correlated [18].

WNT8A is a member of the WNT protein family and plays a crucial role in regulating various biological processes through its interaction with the Wnt/β-catenin signaling pathway. It is particularly important in embryonic and neural development [19,20]. In the early developmental stage of the nervous system, *Wnt8a* is highly expressed and promotes neural tube closure and the differentiation of neural progenitor cells. This process is essential for the normal development of the central nervous system [21]. During regionalization of the nervous system, *Wnt8a* defines the midbrain–hindbrain boundary by inhibiting *Otx2b* and promoting the activation of *Gbx1* [22]. It also participates in the regulation of neural plate gene expression [23]. Thus, the role of *Wnt8a* is instrumental during the patterning of the central nervous system. Nonetheless, the mechanism underlying the transcription regulation of *Wnt8a* is not clear.

In this study, we aimed to study the epigenetic regulation of *Wnt8a* transcription during neurite regrowth of injured rat cortical neurons based on our recent epigenome study.

## 2. Materials and Methods

### 2.1. Antibodies and Reagents

Minimum Essential Medium (MEM), Horse serum (HS), B27 supplement, L-glutamine (L-Gln), Penicillin-streptomycin (P/S), Lipofectamine 2000 Transfection Reagent, TRIzol, MultiScribe™ Reverse Transcriptase (RTase), and Antibiotic-Antimycotic (AA) were purchased from Invitrogen (Carlsbad, CA, USA); Neurobasal medium, Sodium bicarbonate, Sodium Pyruvate, Hank’s Balanced Salt Solution (HBSS), Tryptone, Yeast extract, and Opti-MEM were purchased from Gibco (Grand Island, NY, USA); Poly-L-lysine (PLL), Papain, Cysteine, Calcium chloride (CaCl_2_), Deoxyribonuclease I (DNase I), Glutamate (Glu), Cytosine-β-D-arabinofuranoside (AraC), Sodium chloride (NaCl), Adenosine triphosphate (ATP), Magnesium chloride (MgCl_2_), Chloroform, EGTA, Formaldehyde solution, Sodium butyrate, IGEPAL CA-630, Dithiothreitol (DTT), Hydrochloric acid (HCl), Phenol solution, sodium deoxycholate, Sodium orthovanadate (SV), and kanamycin sulfate were purchased from Sigma-Aldrich (Saint Louis, MO, USA); Triton X-100, EDTA, Sodium dodecyl sulfate (SDS), Sodium acetate, and Phenylmethyl sulfonyl fluoride (PMSF) were purchased from USB (Santa Clara, CA, USA); Fetal bovine serum (FBS) was purchased from PEAK SERUM (Wellington, CO, USA); SYBR Green PCR master mix and High-capacity cDNA reverse transcription kit were purchased from Applied Biosystems (Waltham, MA, USA); Wizard^®^ Genomic DNA Purification Kit was purchased from Promega (Madison, WI, USA); NEBuffer 2.1, NEBuffer 3.1, CutSmart buffer, Nde1, Nhe1-HF, AgeI, Bglll, HindIII, and T4 ligase were purchased from New England Biolabs (Ipswich, MA, USA); Agarose, proteinase K and Glycine were purchased from Chumeia (Paso Robles, CA, USA); NucleoSpin Gel and PCR Clean-up was purchased from MACHEREY-NAGEL (Düren, Köln, Germany); Tris-HCl, and isopropanol were purchased from Avantor (Radnor, PA, USA); Taq DNA polymerase was purchased from GeneTeks Biosciences (Xinbei, Taiwan); 2.5 mM dNTP was purchased from Genezyme biotech (Miaoli, Taiwan); 100 mM dNTP was purchased from GeneDireX (Taoyuan, Taiwan); plasmid miniprep kit was purchased from Biokit (Miaoli, Taiwan); American Bacteriological Agar was purchased from condalab (Torrejón de Ardoz, Madrid, Spain); RNase A, DNase and protease-free was purchased from Thermo Scientific (Waltham, MA, USA); glycogen was purchased from Roche (Basel, CH); anti-H3K4me1 (polyclonal) (Cat# ab8895), anti-H3K4me1 (monoclonal) (Cat# ab176877), and rabbit polyclonal IgG (Cat# ab37415) were purchased from Abcam (Cambridge, UK). Anti-α-tubulin (GTX112141) and anti-green fluorescent protein (GFP, GTX113617) were purchased from GeneTex (Trvine, CA, USA). IRDye 680RD goat anti-mouse IgG secondary antibody and IRDye 800CW goat anti-rabbit IgG secondary antibody were purchased from LI-COR Bioscience (Lincoln, NE, USA). The BCA protein assay kit was purchased from Merck (Kenilworth, NJ, USA).

### 2.2. Experimental Animals and In Vitro Cell Culture of Primary Neurons

All experiments were performed following the guidelines of the Laboratory Animal Center of National Tsing Hua University (NTHU). The protocol for the use of animals was approved by the NTHU Institutional Animal Care and Use Committee. Pregnant Sprague-Dawley (SD) rats were purchased from BioLASCO Taiwan Co., Ltd. First, embryonic day 18 (E18) fetuses were extracted from the uterus of SD rats, the skull was cut to access the brain, and the cortex was dissected from the brain. Primary cortical neurons were dissociated and cultured in MEM supplemented with 5% FBS, 5% HS under 37 °C, and 5% CO_2_ conditions according to Liang et al. [24]. This day is referred to as day-in-vitro 0 (DIV0). On DIV1, the medium was replaced with 1% AA, 25 µM Glu, 0.5 mM L-Gln, 2% B27, and 50 units/mL P/S in the neurobasal medium. A total of 1% AraC was added to cortical neurons on DIV2 to inhibit the proliferation of glial cells. On DIV3, the original medium was replaced with 1% AA, 0.5 mM L-Gln, 2% B27, and 50 units/mL P/S in the neurobasal medium. Half of the medium was replaced on DIV7. Cortical neurons were injured by five vertical and five horizontal scraping lines with P200 tips on DIV8 to simulate mechanical damage to the brains, and samples were collected on DIV9, 10, and 11.

### 2.3. Isolation of Total RNAs, Genomic DNA from Cortical Neurons for Polymerase Chain Reaction (PCR), Reverse Transcription PCR (RT-PCR), and DNA Electrophoresis

Total RNA (1 × 10^7^ cells) was isolated from cortical neurons on DIV9, 10, and 11 via TRIzol according to the manufacturer’s protocol. Genomic DNA extraction from primary neurons was performed according to the manufacturer’s protocol using a Wizard^®^ Genomic DNA purification kit. Reverse transcription (RT) was performed to convert RNA to cDNA using a high-capacity cDNA reverse transcription kit. The RT program was 10 min at 25 °C, 37 °C for 2 h, and 85 °C for 5 min using a Bio-Rad T100 thermal cycler (1861096). PCR was used to amplify cDNA elements followed by analysis on a 1.5% agarose gel to examine the performance of enhancer RNAs in the predicted enhancer regions or the interactions between chromatin conformation capture (3C) products. Taq DNA polymerase was used for the PCR amplification reactions (20 µL) containing 2 µL DNA samples. The thermal cycling conditions included 5 min at 95 °C. Thermal cycling proceeded with 31 cycles of 95 °C for 45 s, 60 °C for 1 min, and 72 °C for 1 min. Then, we incubated the samples at 72 °C for 5 min. In 3C-PCR, only the annealing temperature was changed to 58 °C and the cycle number to 34. The primer sequences used for PCR and RT-PCR are listed in Table S1.

### 2.4. Chromatin Immunoprecipitation (ChIP) and ChIP-Quantitative PCR

For H3K4me1 ChIP, cortical neuron cells (1 × 10^7^) were cross-linked in 0.8% formaldehyde for 10 min at room temperature on DIV9, iDIV9 (injured DIV), DIV10, and iDIV10, then added with 125 mM glycine to stop fixation. A total of 250 µL of lysis buffer was added to the samples followed by sonication for producing DNA fragments. Anti-H3K4me1 or anti-IgG antibodies and protein A beads were added subsequently overnight at 4 °C. On the next day, supernatants were diluted 10-fold in RIPA buffer and transferred to IgG/H4K4me1-bead conjugates for immunoprecipitation before washing and elution. The supernatant was collected and eluted overnight at 65 °C. The samples were treated with RNase A, incubated with proteinase K, and DNA was purified by phenol-chloroform extraction. ChIP-quantitative PCR was performed after ChIP with specific primers. The primer sequences used for qPCR are listed in Table S1.

### 2.5. Chromatin Conformation Capture (3C) Assays

For chromatin conformation capture, the procedure for collecting cortical neuron cells (1 × 10^7^) is the same as ChIP mentioned earlier, and samples were collected at DIV9, iDIV9, DIV10, and iDIV10. First, the sample was lysed with 250 µL RIPA buffer and incubated on ice for 15 min. Then, it was centrifuged at 13,000 rpm for 5 min at 4 °C, we removed the supernatant, and we added a 10-fold diluted r2.1 10× NEB buffer (10 µL was taken as integrity control). Next, the proteins were denatured by adding 0.3% of SDS and shaking at 37 °C, 200/120 rpm for 35 min each. Then, 1.6% Triton X-100 was added to the sample and it was incubated at 37 °C, 200/120 rpm for 35 min each to quench SDS. Afterward, 50 µL samples were collected as undigested control. HindIII was added to the samples and they were incubated at 37 °C, 200 rpm for 2 h, then at 37 °C, 120 rpm overnight to digest the sample (take 50 µL sample as digested control). On the next day, we added 1.4% SDS to quench the restriction enzyme and incubated the samples at 65 °C, 120 rpm for 30 min. We added the samples to 5.6 mL of ligation buffer and 0.001 M ATP to quench SDS with 1% Triton-X-100, and incubated them at 37 °C, 200 rpm for 60 min. To ligate fragmented DNA elements, T4 DNA ligase was added to the samples and they were incubated overnight at 4 °C. Samples were then treated with proteinase K overnight to degrade proteins and RNase A to degrade RNA. An equal amount of phenol-chloroform was added to the sample to isolate the DNA. The supernatant was collected after centrifugation at 13,000 rpm for 15 min at 4 °C; then, 0.06 M NaCl, 2.5 μg/mL glycogen, and 18 mL absolute ethanol were added to precipitate DNA at −80 °C for 1 h. The samples were centrifuged and pellets were dissolved with 1 mL ddH_2_O at 60 °C for 1 h before storing at −20 °C.

### 2.6. Complementary Digestion

Additional digestion could be performed after 3C to enhance the results of 3C-PCR. Add 20 µL of 10× NEBuffer 3.1 and 100U BglII (not present in the ligated sample) to 150 µL of 3C product; then, make up to 200 µL with ddH2O and incubate for 2 h in a 37 °C water bath. Add 200 µL of phenol-chloroform for the isolation of DNA from proteins and lipids. After centrifugation at 13,000 rpm for 5 min at 4 °C, add 20 µL of 2 M sodium acetate (pH 5.6) and 500 µL of absolute ethanol to the supernatant; then, incubate at −80 °C for 45 min. Centrifuge at 13,000 rpm at 4 °C for 20 min would be performed afterward. Finally, the samples were washed with 500 µL 70% ethanol and dissolved in 150 µL ddH_2_O. The additional digested sample was stored at −20 °C.

### 2.7. Enhancer–Reporter Assays

The pEGFP-C2 plasmid was digested with AgeI and Nhe1-HF, replacing the CMV promoter part of the backbone with the rat *Wnt8a* promoter forming the pEGFP-C2-*Wnt8a* promoter. Then, the En8 region, En14 region, and the random sequences (as a negative control) were inserted into the pEGFP-C2-*Wnt8a* promoter using Nde1 and Nhe1-HF, respectively. A total of 7.5 × 10^5^ Neuro2a (N2A) cells, a mouse neural crest-derived cell line, were seeded on the 10 cm culture dishes for 24 h at 37 °C. After 24 h of cell culture, the medium of N2A cells was replaced with Opti-MEM. A total of 2 mL of mixtures containing 10 µg of reporter plasmid and 20 µL of lipofectamine were added into the cells for transfection, then they were incubated at 37 °C for 4 h. Afterward, the medium was replaced with the 7 mL original medium of N2A cells. After 48 h, images were recorded with a fluorescence microscope (observer Z1, ZEISS). The fluorescence intensity of EGFP was quantified by ImageJ (version 1.54p) and determined as corrected total cell fluorescence (CTCF) = integrated density − (area of selected cell × mean fluorescence of background reading). Each CTCF value was normalized to the mean EGFP CTCF of the N2A cells transfected with the pEGFP-C2-Random sequences-*Wnt8a* pro.

### 2.8. Western Blotting

For Western blot analysis, proteins were collected from N2A cells after 48 h transfection with 100 µL lysis buffer. The samples were run on a 12% SDS-PAGE, followed by transfer onto a nitrocellulose membrane. The membrane was blocked with 1% BSA for 1 h and incubated with anti-GFP (1:5000) or anti-α-tubulin (1:1000) antibodies overnight at 4 °C under rotation. After that, the IRDye 680RD goat anti-mouse IgG secondary antibody (1:10,000) and IRDye 800CW goat anti-rabbit IgG secondary antibody (1:10,000) were used as the secondary antibodies. The protein expressions were detected by LI-COR ODYSSEY.

### 2.9. ENCODE Dataset Processing

ChIP-seq data for H3K4me1 and CTCF of *Mus musculus* were collected from the ENCODE database. All H3K4me1 ChIP-seq datasets of mouse forebrain were extracted from ENCFF739NQW (E10.5, GEO: GSE86779), ENCFF409EOT (E13.5, GEO: GSE82738), ENCFF190CPH (E16.5, GEO: GSE68507), and ENCFF326LFP (P0, GEO: GSE82717). The binding pattern of CTCF on P0 was extracted from ENCFF500SSQ (GEO: GSE91978). RNA expression in CNS during development was also obtained from the ENCODE database (BioProject: PRJNA66167).

### 2.10. Data Analysis

The results of PCR followed by DNA electrophoresis were analyzed with the Gel-Pro Analyzer 3.1 software, and the integrated optical density values of the samples were recorded and normalized to the control. The results of the enhancer RNA were normalized to GAPDH, and the 3C sample was normalized to the loading control. The results of the ChIP-qPCR were normalized to the tenfold dilution of the input. The enrichment of histone modification was calculated as a percentage of input (% input) = 100% × 2^{−[Ct(ChIP) − Ct(input)]}^. The value of Ct was calculated by the StepOne software v2.3 during the process of qPCR. The results of the reporter assay were analyzed by western blot and normalized to the α-tubulin.

### 2.11. Statistical Analysis

All results were presented as the mean ± standard error of the mean (S.E.M.) of at least three independent experiments. The two-tailed student’s *t*-test or one-way ANOVA were used for statistical analysis. *p* values < 0.05 were considered significant.

## 3. Results

### 3.1. Identification of Enhancer Region for Wnt8a Transcription During Neurite Regrowth of Injured Cortical Neurons

**Figure 1:**
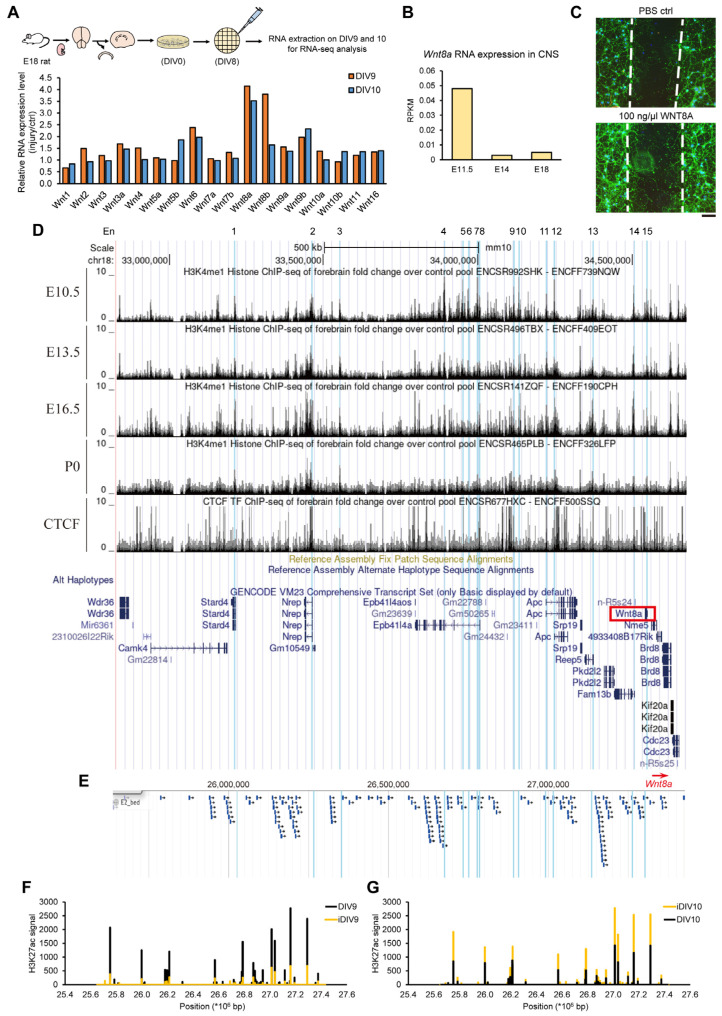
Identification of enhancer regions for *Wnt8a* gene. (**A**) The expression levels of the WNT gene family were analyzed by RNA sequencing. RNA samples were collected from three batches of cortical neurons on DIV9 (orange) and DIV10 (blue). Three batches of RNA samples were combined with an equal amount for subsequent RNA-seq analysis. The expression level of the injury group was normalized to the non-injury control group on DIV9 and DIV10, respectively. (**B**) The RNA expression of *Wnt8a* on E11.5, E14, and E18 of CNS in a C57BL/6J mouse (mm10). Data were obtained from the ENCODE database (BioProject: PRJNA66167), which comprised two biological replications. (**C**) Primary cortical neurons were isolated from E18 rat brains and cultured in vitro; day in vitro 0 = DIV0. On DIV8, cortical neurons pretreated with PBS (vehicle control) or 100 ng/μL WNT8A recombinant protein for 1h were scraped and injured by p20 tips. Cortical neurons were fixed for immunostaining on iDIV11. DAPI (blue) was used to label the nucleus. Anti-TUJ1 antibody (green) was used to visualize neurite regrowth. Fluorescence pictures were taken using a Carl Zeiss Observer Z1 microscope. White dashed lines indicate the border of the injury gap. Scale bar = 100 μm. (**D**) The signals of H3K4me1 modifications upstream of *Wnt8a* on E10.5 (ENCFF739NQW), E13.5 (ENCFF409EOT), E16.5 (ENCFF190CPH), and P0 (ENCFF326LFP) from the ENCODE database, which has two biological replications. The predicted enhancer regions, from En1 to En15, were labeled as blue highlights. (**E**) Predicted enhancer signal showed in JBrowse Genome Browser (blue arrow). The region started from 25,633,520 to 27,428,616 on rat chromosome 18, where *Wnt8a* is located. (**F**,**G**) The signal of the H3K27ac modification upstream of *Wnt8a*. The samples were collected from cortical neurons on DIV9, iDIV9, DIV10, and iDIV10 for ChIP-seq analysis. The non-injury groups were labeled as black, and the injury groups were labeled as yellow.

To examine the neurite regrowth of injured cortical neurons, we have established an in vitro TBI model to monitor the process of neurite regrowth. For this, embryonic day 18 (E18) cortical neurons derived from Sprague-Dawley (SD) rat brains were cultured on day-in-vitro 0 (DIV0). On DIV8, cortical neurons were mechanically injured via scraping, and neurite regrowth was monitored for 48 h. RNAs were collected 24 h and 48 h after injury because neurite regrowth was found to be most robust between 24 and 48 h in vitro [25]. We performed whole genome chromatin immunoprecipitation-sequencing (ChIP-seq) and RNA-sequencing (RNA-seq) analysis on cortical neurons 24–48 h after injury versus non-injury controls [26]. We have, thus, identified a number of candidate regeneration-associated genes (RAGs), including a panel of 18 WNT genes (Figure 1A). Among these WNT genes, the relative expression of the *Wnt8a* gene during neurite regrowth was the highest, 3.5- to 4.1-fold compared to the non-injury controls, based on RNA-seq results. This high induction could probably be due to reduced basal level in the late embryonic stage, as evidenced by ENCODE data of *Wnt8a* during the developmental stage from E11.5, 14 to 18 (Figure 1B). Upon injury, the transcriptional induction of *Wnt8a* may serve to promote the regeneration process. To determine the functional outcome, the WNT8A recombinant protein was added to the culture of injured cortical neurons and the neurite regrowth was imaged. The results showed that the WNT8A protein can promote neurite regrowth in injured cortical neurons, as seen in Figure 1C and Figure S1 and in the study by Chang et al. [25].

Enhancer RNAs transcribed from active enhancers are significant regulators of the epigenome. In response to brain injury, epigenome remodeling governs the success of regeneration of injured neurons. We used a previously established algorithm [26] to predict candidate enhancer regions of the *Wnt8a* gene. Briefly, ChromHMM was used for chromatin-state discovery on the basis of chromatin modification patterns by model training and analysis. Published H3K4me3 (active promoter mark), H3K27ac (active enhancer mark), and RNAPII Chip-seq datasets were used for model training. Genomic elements with highly enriched H3K27ac modification and low occupancy of RNAPII that are distal to the transcription start site (TSS) are likely to feature enhancer regions. Based on ChromHMM analysis, regions from 1.7 Mb upstream to 0.1 Mb downstream were predicted as candidate enhancer regions. The regions showed higher H3K4me1 (active enhancer mark) during embryonic stage E16.5 and decreased on postnatal stage P0 in CNS suggestive of a high *Wnt8a* expression in mice during CNS development then gradually decreased before birth (Figure 1D). Some of these regions were within the enhancer regions that we predicted based on H3K27ac enrichment and low RNA polymerase II occupancy, concomitant with the lack of H3K27ac and RNA polymerase II occupancy proximal to the promoter (Figure 1E). Among these regions, those with increased H3K27ac signals during neurite regrowth were identified (Figure 1F,G). Based on these selection criteria, the candidate enhancer region of *Wnt8a* was further subdivided into En1–En15 regions (Figure 1D), each of which was 600 bp long. Since the predicted enhancer regions span a long distance that may include other genes, H3K4me1 and H3K27ac marks could potentially be enhancers for other promoters or enhancers of genes proximal to *Wnt8a*. To this end, the RNA-seq data of *Nme5*, *Brd8*, *Fam13b*, *Apc*, *Srp19*, *Reep5*, *Epb4114a*, *Nrep*, *Stard4*, *Pkd212*, *Camk4*, and *Kif20a* genes were examined on DIV9, iDIV9 (injured DIV9), DIV10, and iDIV10 (injured DIV10). Except for *Wnt8a*, the expressions of these genes did not change during neurite regrowth (Figure S2). These results suggest that the predicted enhancer region is most likely responsible for transcriptional regulation of the *Wnt8a* gene.

### 3.2. Subsets of Enhancer RNAs Are Increased During Neurite Regrowth

**Figure 2:**
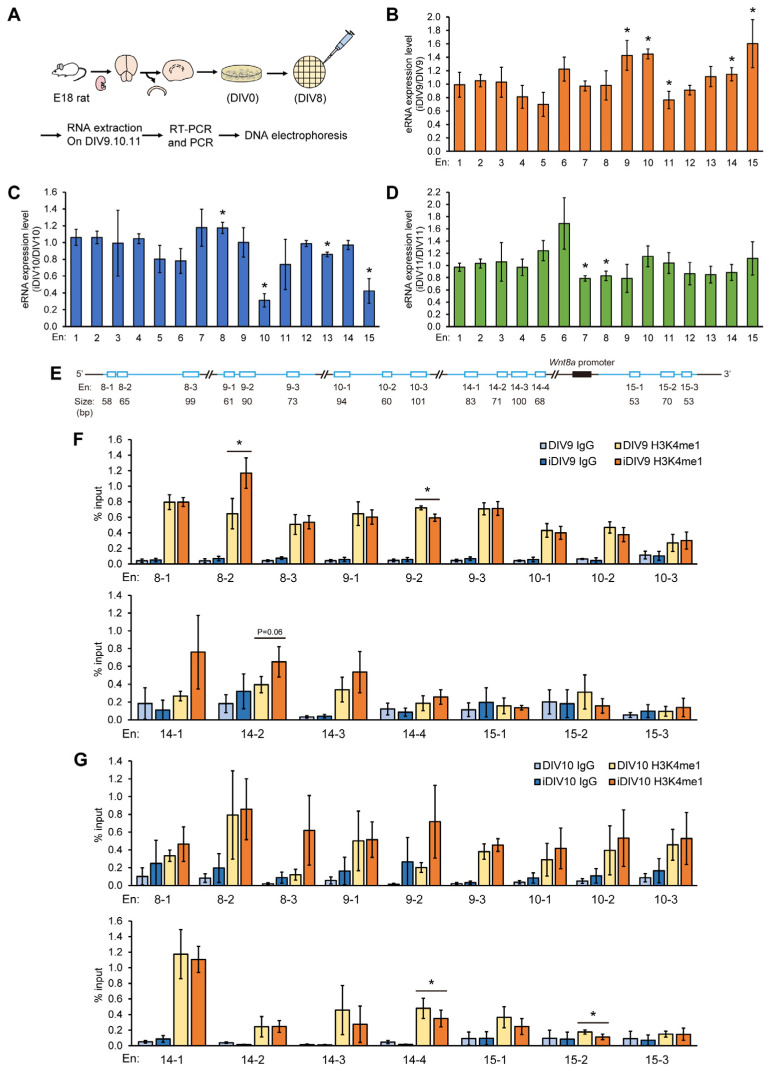
The eRNA levels and H3K4me1 modifications of the putative *Wnt8a* enhancer region during neurite regrowth. (**A**) Schematic overview of the experimental design for investigating expressions of eRNAs. The putative enhancer region is divided into 15 subregions (from En1 to En15). The expression of eRNAs from En1 to En15 on DIV9 (**B**), DIV10 (**C**), and DIV11 (**D**) were amplified and analyzed by DNA gel electrophoresis during neurite regrowth. eRNA levels were normalized to non-injury groups. Data were presented as mean ± SD from at least three independent experiments. (**E**) Schematic diagram of enhancer subregions for Chip-qPCR assays. (**F**,**G**) ChIP-qPCR data of the H3K4me1 signals at En8, En9, En10, En14, and En15 on DIV9 and DIV10. Each enhancer region was divided into 3–4 subregions for subsequent analysis. Enrichment was normalized to input. IgG was used as a negative control. Data were presented as mean ± S.E.M. from at least three independent experiments. Statistical analysis was performed using the Student’s *t*-test. * *p* < 0.05.

In addition to marking the active enhancers, eRNAs could assist gene expression by promoting promoter-enhancer looping, enhancing interaction between eRNAs and transcription factors, and facilitating H3K27 acetylation. To investigate the activity of predicted enhancer regions of *Wnt8a* during neurite regrowth, the expressions of eRNAs from the predicted enhancer regions on DIV9, DIV10, and DIV11 after injury were examined. Cortical neurons from E18 rats were obtained and cultured till DIV8. Neurons were scraped on DIV8 and RNAs were collected on DIV9, 10, and 11 for RT-PCR analysis using specific primer sets (Figure 2A). There was no significant difference between control and injury eRNAs derived from the En1–En6 regions during DIV9–11 (Figure 2B–D). On DIV9, eRNAs from the En9, 10, 14, and 15 regions were induced significantly during neurite regrowth (Figure 2B). The level of eRNA expression from the En8 region was increased on iDIV10 compared to DIV10 (Figure 2C). On the contrary, the expression levels of eRNAs from the En11 region on iDIV9, the En10, 13, and 15 regions on iDIV10, and the En7 and En8 regions on iDIV11 declined significantly during neurite regrowth. Collectively, these results suggest that the En8, 9, 10, 14, and 15 regions could be the active enhancer regions of *Wnt8a* during neurite regrowth [25,26]. To examine the enhancer-associated histone modifications during regeneration, chromatin immunoprecipitation (ChIP) experiments were performed using the anti-H3K4me1 antibody on lysates derived from cortical neurons on DIV9, iDIV9, DIV10, and iDIV10, followed by qPCR analysis with specific primers (Table S1). The En8, 9, 10, 14, and 15 regions were further classified into three to four subregions for the ChIP experiments. H3K4me1 signals of En8-2 and En14-2 regions were significantly increased on iDIV9 and slightly increased at the En14-1 and En14-3 regions during regrowth (Figure 2E). H3K4me1 modification was significantly reduced in the En9-2 on iDIV9 and the En14-4 and En15-2 regions on iDIV10 (Figure 2F). These results reveal that both En8-2 and En14-2 participate in the regulation of *Wnt8a*, possibly through the dynamic interaction of these two subregions.

### 3.3. Enhancer–Promoter Interplay for Wnt8a Transcription During Neurite Regrowth

**Figure 3:**
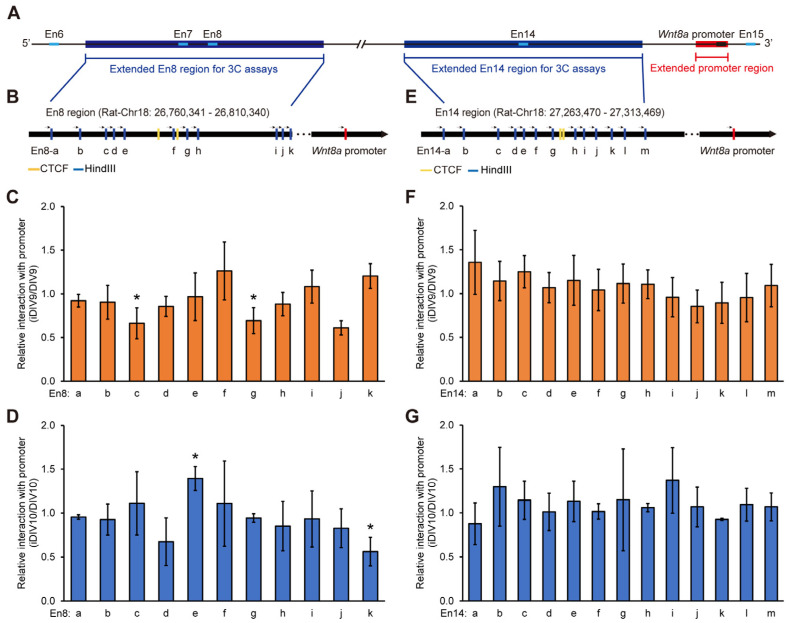
The interaction between En8, En14, and the promoter of *Wnt8a,* as determined by Chromatin Conformation Capture (3C) assays. (**A**) Schematic diagram of extended En8 (blue), En14 (blue), and *Wnt8a* promoter (red) regions for 3C assays. (**B**) Schematic diagram of En8 and En14 (**E**) subregions based on HindIII restriction cutting sites (blue). CTCF binding sites were marked in yellow and the *Wnt8a* promoter was marked in red. The interaction relationship between En8 and the *Wnt8a* promoter was analyzed on DIV9 (**C**) and DIV10 (**D**). The interaction between En14 and the *Wnt8a* promoter was detected on DIV9 (**F**) and DIV10 (**G**). The quantified results of DIV9, iDIV9, DIV10, and iDIV10 were normalized to the control (without HindIII cutting site), respectively. The ratio of the injury group (iDIV9 and iDIV10) to the non-injury group (DIV9 and DIV10) served as a relative interaction. Data are presented as mean ± S.E.M. from at least three independent experiments. Statistical analysis was performed using the Student’s *t*-test. * *p* < 0.05.

To access the genomic interaction and the interplay among En8, En14, and the promoter regions of *Wnt8a*, chromatin conformation capture (3C) assays were established [27,28,29]. A 3C-PCR was performed to detect their potential interaction. Due to the limited choice of the restriction endonuclease cleavage site around these regions, we tested 50,000 bp extended regions beyond the En8 and En14 regions (Figure 3A). A HindIII restriction enzyme was used to digest DNA around these regions and directional primers were designed to assess the putative interaction between the subregions from En8-a to En8-k and the *Wnt8a* promoter (Figure 3B). The physical proximity of DNA fragments was assessed by 3C-PCR, and amplification was only possible in the presence of chromatin interactions between these distant DNA fragments. The relative interaction between the En8-c, En8-g, and *Wnt8a* promoters was decreased on iDIV9 (Figure 3C). During neurite regrowth, the interaction level between the *Wnt8a* promoter and the En8-e subregion was increased significantly (Figure 3D), whereas it was decreased with the En8-k subregion on iDIV10. The subregions of the En14 region are shown in Figure 3E. There was no significantly different interaction between the En14 subregions and *Wnt8a* promoter both on iDIV9 and iDIV10 (Figure 3F,G).

**Figure 4:**
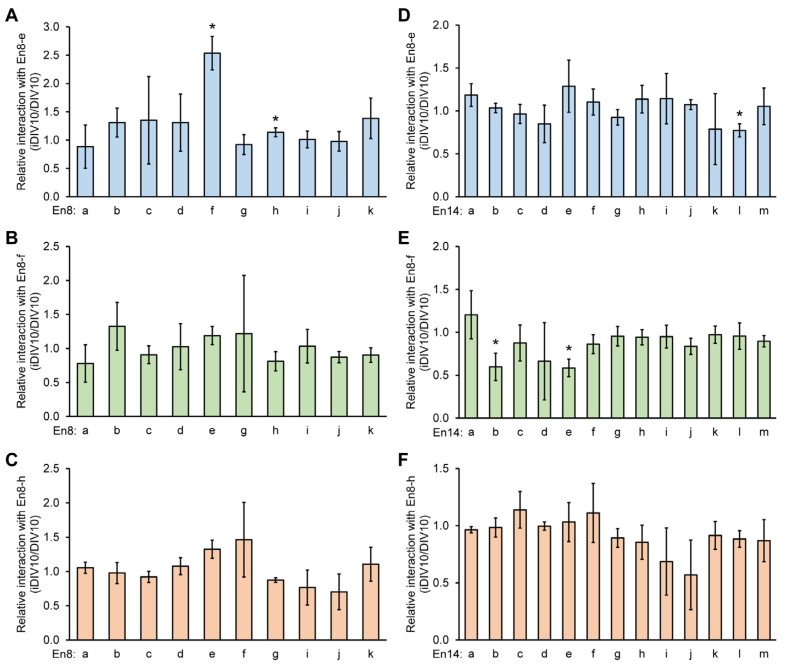
Relative interaction between subregions of En8 (En8-a to En8-k) and En14 (En14-a to En14-k) by 3C assays. On DIV10, 3C samples were collected from cortical neurons. The sample was digested by the HindIII restriction enzyme for 3C assays. The 3C-PCR product without the HindIII cutting site served as the control group. (**A**) The genomic interactions among subregions of En8 and En8-e, or with En8-f (**B**), or with En8-h (**C**). (**D**) The interactions between subregions of En14 and En8-e, or with En8-f (**E**), or with En8-h (**F**). The quantified data were normalized to the control (without the HindIII cutting site). The ratio of the injury group to the non-injury group served as a relative interaction. Data are presented as mean ± S.E.M. from at least three independent experiments. Statistical analysis was performed using the Student’s *t*-test. * *p* < 0.05.

To further analyze the regulation network of *Wnt8a* through the En8 subregions, the interactions of En8-e with the other En8 subregions were also examined. The data showed that the interactions among the En8-e, En8-f, and En8-h subregions were significantly increased during neurite regrowth (Figure 4A). As En8-f and En8-h contain no obvious interaction within the En8 region during neurite regrowth (Figure 4B,C), the interactions between the En8-e subregion and En14-l subregion were significantly reduced on iDIV9 compared to DIV9 (Figure 4D). The interactions of En8-f with the En14-b or En14-e subregion were significantly decreased on iDIV10 compared to DIV10 (Figure 4E). On the other hand, there was no interaction between the En8-h and En14 subregions during neurite regrowth (Figure 4F).

### 3.4. En8 and En14 Enhance Wnt8a Promoter Activity

**Figure 5:**
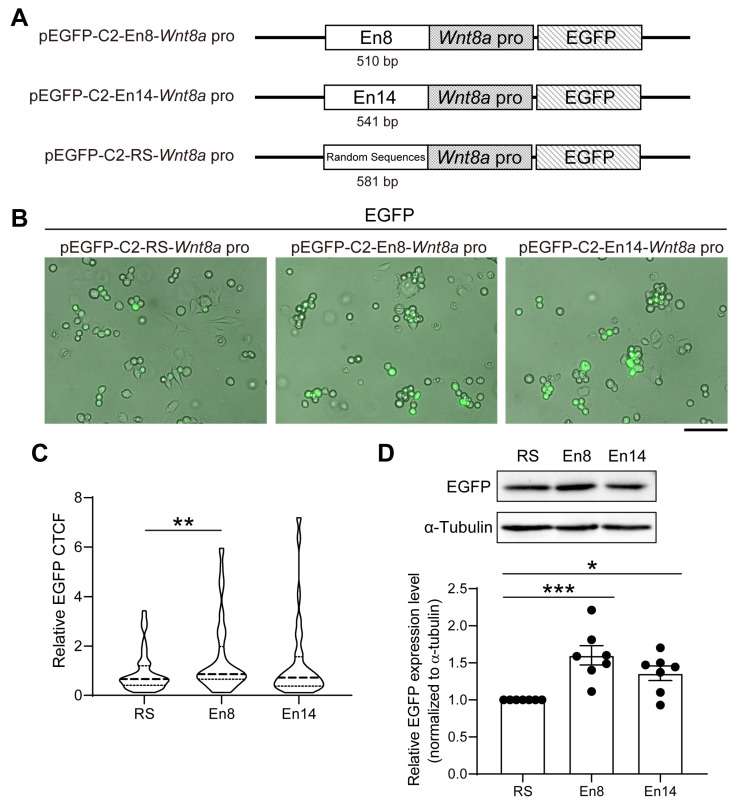
Potential collaboration between En8, En14, and promoter to drive transcription of *Wnt8a*. (**A**) Three reporter constructs were engineered: (1) pEGFP-C2-Random Sequences (RS)-*Wnt8a* pro containing *Wnt8a* promoter that is fused to GFP; En8 (2) or En14 (3) is inserted to 5′ of promoter sequences. (**B**) N2A cells were transiently transfected with pEGFP-C2-RS-*Wnt8a* pro, pEGFP-C2-En8-*Wnt8a* pro, or pEGFP-C2-En14-*Wnt8a* pro. GFP fluorescence was overlaid on the bright field image 48 h after transfection. The scale bar represents 100 μm. (**C**) The corrected total cell fluorescence (CTCF) of EGFP in transfected N2A was quantified. The EGFP fluorescence intensity was measured by ImageJ and normalized to the mean EGFP CTCF of N2A cells transfected with pEGFP-C2-RS-*Wnt8a* pro. At least fifty cells were quantified from each experiment group. (**D**) Lysates were collected from (**B**) for subsequent Western blotting with anti-GFP and α-tubulin antibodies. The relative EGFP protein levels were quantified and normalized to α-tubulin loading control and then to that of pEGFP-C2-RS-*Wnt8a* pro. Data were presented as mean ± S.E.M. from at least three independent experiments. Statistical analysis was performed using the one-way ANOVA. * *p* < 0.05, ** *p* < 0.01, *** *p* < 0.001.

While the 3C experiments revealed the physical interactions between the *Wnt8a* promoter and the candidate enhancers, they did not address the function and relative efficacy of the enhancer–promoter on the transcriptional activity of *Wnt8a*. To this end, we constructed the enhancer and promoter reporter constructs, pEGFP-C2-Random sequences (RS)-*Wnt8a* pro, pEGFP-C2-En8-*Wnt8a* pro, and pEGFP-C2-En14-*Wnt8a* pro as shown in Figure 5A. If En8 and En14 interact with the promoter to drive *WNT8a* transcription, EGFP fluorescence intensity will report the relative efficiency of the enhancer–promoter. These constructs were transiently transfected to neuroblastoma-derived N2A cells for 48 h, and EGFP fluorescence was determined via fluorescence microscopy (Figure 5B). The EGFP intensity of N2A cells transfected with pEGFP-C2-En8-*Wnt8a* pro was significantly increased (Figure 5C). In addition, the relative expression levels of EGFP were also quantified by western blotting. N2A cells transfected with both pEGFP-C2-En8-*Wnt8a* pro and pEGFP-C2-En14-*Wnt8a* pro showed a significantly increased EGFP expression compared to that of transfected with EGFP-C2-RS-*Wnt8a* pro (Figure 5D).

**Figure 6:**
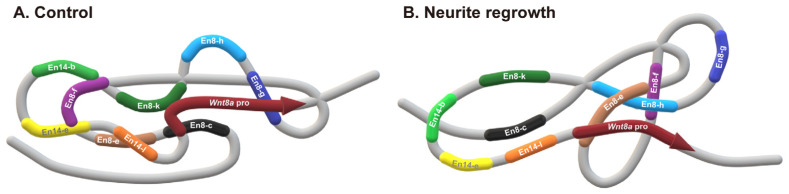
A putative model of enhancer–promoter-mediated *Wnt8a* expression during neurite regrowth. (**A**) At the resting state, the *Wnt8a* promoter (red) interacted with the En8-c (black), En8-g (blue), and En8-k (green) regions. The En8-f (purple) interacts with both En14-b (light green) and En14-e (yellow), whereas En8-e (brown) interacts with the En14-l (orange). (**B**) During neurite regrowth, the *Wnt8a* promoter (red) interacts with En8-e (brown), which concomitantly binds to En8-f (purple) and En8-h (light blue), to facilitate transcription of *Wnt8a* during neurite regrowth. Microsoft Paint 3D software (version 6.2410.13017.0) was used to create this model.

Subregions En8-c, En8-g, and En8-k interact with the *Wnt8a* promoter, while the En8-e subregion interacts with En14-l, and both the En14-b and En14-e subregions interact with the En8-f subregion at the resting stage (Figure 6A). After a neuronal injury, the En8-e could interact with the En8-f and En8-h subregions and activate the *Wnt8a* gene expression by interacting with the *Wnt8a* promoter. Based on these findings, we propose a regulatory mechanism for *Wnt8a* transcription during neurite regrowth as illustrated in Figure 6B.

## 4. Discussion

While eRNAs transcribed from the En8 and En14 regions, as well as the H3K4me1 and H3K27ac modifications, were increased during neurite regrowth, the interaction between the *Wnt8a* promoter and En14 region was undetected in the 3C experiment. It is possible that En14-generated eRNAs serve to recruit regulators and transcription factors and to promote the acetylation of H3K27 for subsequent activation of *Wnt8a* expression. On the other hand, En8 region loops interact directly with the *Wnt8a* promoter and may serve to bridge another layer of transcriptional regulation in combination with co-activators. Both the En8 and En14 regions can be modified by H3K4me1 to remodel nucleosomal structures to improve chromatin accessibility. The nuclei of eukaryotic cells spatially separate chromosomes to form basic units of three-dimensional nuclear organization called topologically associated domains (TADs), which play a key role in maintaining transcriptional regulation. TADs are identified by chromosome conformation capture techniques, including Hi-C, and the boundaries between TADs contributed to restricting abnormal interactions of cis-regulatory sequences to their target genes. To investigate the intrachromosomal interaction between the *Wnt8a* promoter and the En8-e subregion, the Hi-C data were examined on juicebox online tools [https://aidenlab.org/juicebox/ (accessed on 18 August 2022)]. Data on rat cortical tissue are not available in juicebox; thus, the interactions between the En8-e subregion (Chr18: 33,999,159–34,004,158) and the *Wnt8a* promoter (Chr18: 34,538,368–34,543,368) in mouse neural progenitor cells (NPCs) were examined. Consistent with our finding, there were no obvious interactions between the En8-e region and the *Wnt8a* promoter at the resting state, suggesting a low-to-none *Wnt8a* expression at the late stage of neuronal differentiation (Figure S3). During neurite regrowth, En8-e and promoter interaction are likely to re-initiate *Wnt8a* expression based on our 3C data.

Our previous ChIP-sequencing analysis identified WNT genes as candidate RAGs by examining histone H3K27ac modifications. Recently, we reported that H3K4me1 modifications within enhancers play a decisive role in driving the transcription of the *Wnt3a* gene [26]. Consistently, we observed an increased H3K4me1 modification within the En8 region for the *Wnt8a* gene on iDIV9 in the current study. These findings suggest that H3K27ac and H3K4me1 have distinct roles in gene regulation. Histone H3K27ac is often associated with active enhancers and promoters, acting by reducing the positive charge on histones and their interaction with the negatively charged DNA, thus leading to a more open chromatin structure and facilitating transcription factor binding and transcriptional activation. H3K4me1 modification, on the other hand, is commonly found in enhancers, both those that are active and those that are poised to become active. Through the looping mechanism, H3K4me1 modification can be further methylated into H3K4me3 and associated closely with active promoters. Together, these histone modifications contribute to the dynamic regulation of gene expression, orchestrating the preparatory modification and activation of genes in response to injury cues.

## 5. Limitation of This Study

The experiments do not fully consider all conditions that can appear in real situations. Hence, caution should be taken with generalizing the findings and applying them to real-life situations. The culture of cortical neurons was derived from a cortex that contained a number of neuron types. For each brain dissection and culturing, it is possible that a certain neuron type was enriched and thus contributed to the observed data variation. In addition, the low percentage of injury used in the primary neuronal culture also contributed to the under-estimated injury response and the increase in RAGs during neurite re-growth. Moreover, we acknowledge that the percentage of interaction among subregions of the genome is, in general, very low by nature, and, as such, data variation is obvious in certain data points. A larger sample number may further validate the data.

In future research, it would be helpful to further investigate the roles of other members of the WNT gene family in neuronal regeneration and compare their similarities and differences with *Wnt8a*. Furthermore, based on a comprehensive analysis of gene regulatory networks, we could explore how different transcription factors and coactivators are involved in the regulation of WNT genes, especially their roles in the early injury response. These studies will help us gain a deeper understanding of the molecular mechanisms of neuronal regeneration and may provide new therapeutic targets and strategies for clinical treatment. Recombinant protein technology is also widely used in the biomedical field, especially in the development of therapeutic drugs and vaccines [30]. The most classic example of the use of recombinant insulin to treat type 1 diabetes (T1D) was proposed by Herbert Boyer et al. It has become a major therapy and continues to be in use to this day [31]. Our study suggests that *Wnt8a* acts as an effective RAG to promote neuronal regeneration. In clinical practice, WNT8A recombinant protein therapy will be another potential treatment approach. To this end, more animal studies are needed to evaluate the effects of WNT8A recombinant protein treatment in an in vivo brain injury model.

## 6. Conclusions

In this study, we predicted candidate enhancer regions regulating *Wnt8a* transcription during neurite regrowth based on ChIP-seq and RNA-seq analysis. This study has identified the enhancer for the *Wnt8a* gene and uncovered the novel transcriptional regulation of the *Wnt8a* gene during neurite regrowth. Our findings highlighted the contribution of H3K4me1 modification in identifying the active enhancer region which, in turn, orchestrates the enhancer–promoter interplay to increase the expression of *Wnt8a*. These results not only enhance our understanding of the regulation of gene expression after neuronal injury but also provide new perspectives for neural repair.

## Data Availability

The data presented in this study are available on request from the corresponding author.

## Supplementary Materials

The following supporting information can be downloaded at: https://www.mdpi.com/article/10.3390/cells14050319/s1, Figure S1: WNT8A recombinant protein treatment promoted injured neurite regrowth of cortical neurons. Figure S2: The relative expressions of genes upstream of *Wnt8a* based on RNA-seq data. Figure S3: Intrachromosomal interaction between *Wnt8a* promoter (Chr18: 34,538,368–34,543,368) and En8-e region (Chr18: 33,999,159–34,004,158) of mouse Hi-C data. Figure S4: Individual immunoblots of EGFP and α-Tubulin in reporter assays. Table S1: List of primer sequences for PCR, and qPCR.
