# Supplementary material for: The Enhancer–Promoter-Mediated Wnt8a Transcription During Neurite Regrowth of Injured Cortical Neurons
Source: Cells. 2025 Feb 20;14(5):319. doi: 10.3390/cells14050319 (PMC11898497; doi:10.3390/cells14050319)
Supplement: Supplementary file 1 [file cells-14-00319-s001.zip › cells-3422861-supplementary.pdf]

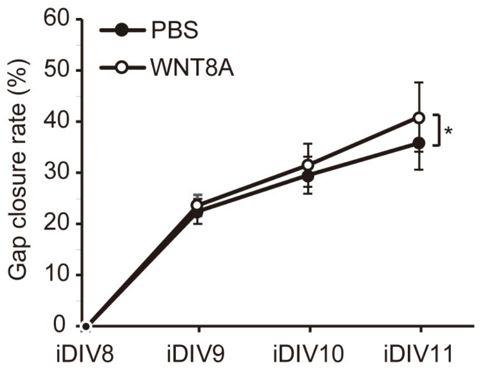

Figure S1. WNT8A recombinant protein treatment promoted injured neurite regrowth of cortical neurons. Primary cortical neurons were isolated from E18 rat brains and cultured in vitro, day in vitro 0 = DIV0. On DIV8, cortical neurons pretreated with PBS vehicle control or 100 ng/μl WNT8A recombinant protein for 1h were scraped and injured by p20 tips (iDIV8). Cortical neurons were fixed for immunostaining on iDIV11. The efficacy of neurite regrowth was evaluated by gap closure rate. The percentage of gap closure was calculated as (1 – length of gap between injured borders/length of gap between regenerated neurites) × 100%. The higher gap closure rate means injured cortical neurons had more regrowing neurites. Data were presented as mean ± S.E.M. from at least three independent experiments.

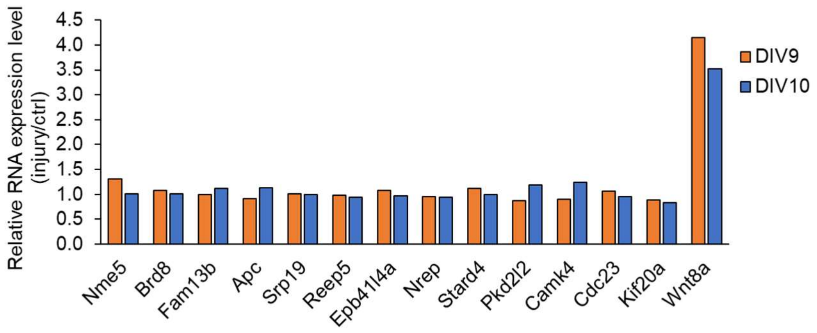

Figure S2. The relative expressions of genes upstream of *Wnt8a* based on RNA-seq data. Total RNA samples were collected from three batches of rat cortical neurons on DIV9 (orange) and DIV10 (blue) for RNA-seq analysis. The expression level of the injury group was normalized to the non-injury control group on DIV9 and DIV10, respectively.

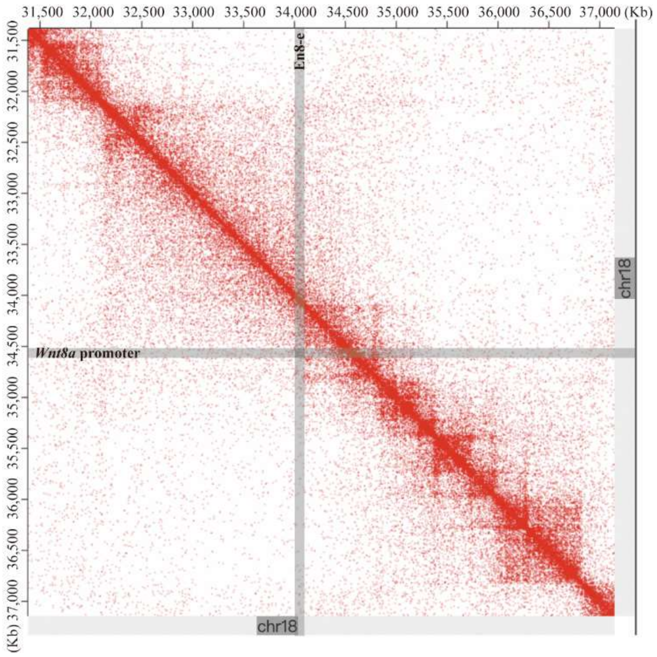

Figure S3. Intrachromosomal interaction between *Wnt8a* promoter (Chr18: 34,538,368–34,543,368) and En8-e region (Chr18: 33,999,159–34,004,158) of mouse Hi-C data. The Hi-C chromatin interaction map of chromosome 18 in neural progenitor cells (NPCs) of mouse. Red indicates more frequent interactions and white indicates no interactions. The dark area indicates the location of the *Wnt8a* promoter and En8-e subregion on chromosome 18.

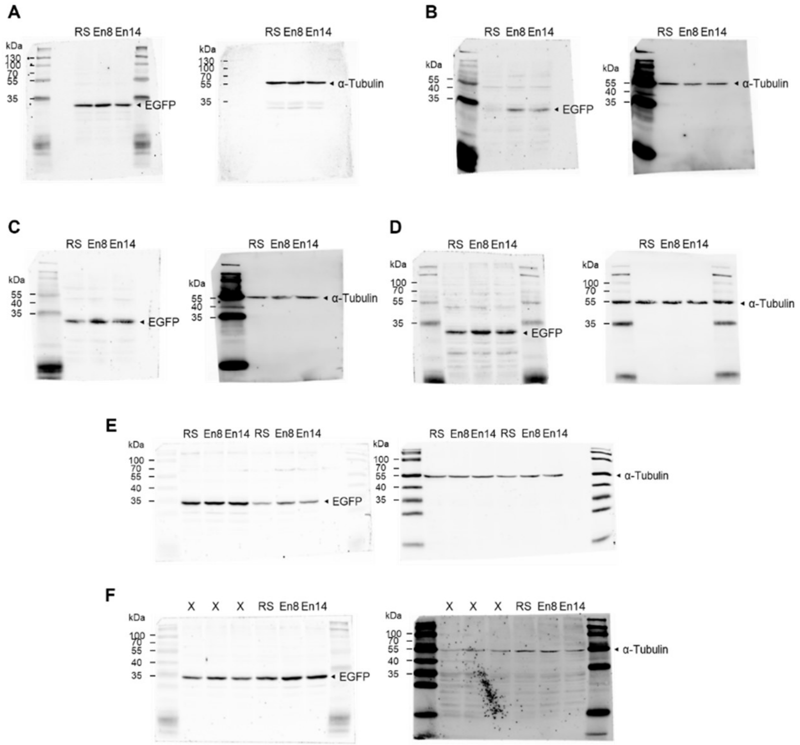

Figure S4. Individual immunoblots of EGFP and α-Tubulin in reporter assays. N2A cells were transiently transfected with reporter constructs, pEGFP-C2-RS-*Wnt8a* pro, pEGFP-C2-En8-*Wnt8a* pro, or pEGFP-C2-En14-*Wnt8a* pro. After 48 h, lysates were collected for subsequent

Western blotting with anti-GFP and α-Tubulin antibodies. Arrows indicate specific bands of EGFP and α-Tubulin.

Table S1. List of primer sequences for PCR, and qPCR

Primers used for enhancer RNA expressions		
Regions	Forward primer (5′-3′)	Reverse primer (5′-3′)
En1	TCCAGGAGTGGGATCGTTGT	TGGAGAAGCTGTTCGTTCGT
En2	GCCTTTGTGGTGTGGCTAGA	AGCACCCGAGTCTTACTTGAC
En3	AGGCCATATGGATGTTTTGCTAGT	GATGGAAATGACAGAGCAAATGAG
En4	CGGGATCCAGCCAATACTGT	TACATGATGATGGCCGGGGAG
En5	CCCGCCAACCAAAACTGAAAG	AAGCATCGTGGGCTGTAAGG
En6	CAGGATTTCCCGTGCTGCTTA	TAGCTGCCAGACTCCAGAAA
En7	TACCGGCTGTGTGGAAATCTG	AGATCCCCAAGGGCTGAAATG
En8	GTCACGGTGGCCTTTCTTCT	ACCATAATCGGTTTGCTCCTGT
En9	TGTTAGCTGTTCCTTCCGGTG	TGCTAGAGTAGTTTTGCTGCCT
En10	GCCTAACCACCAGCCTTTTCT	ATAGCCCCCACTGCCTCTAA
En11	ATGGCACAGGAAAGGATTGC	ACAAGCACTCTACCACTGGG
En12	GGCTCGAAAATGGGGTAGGT	CCATCCTCGGTAGCCCTTTT
En13	ACCCGAGCACCAAGAAAGAA	AAGGGCGATTTCAGGAGTGTT
En14	AATGTCACCACAGTAGACCCT	GGGCGTCAAGTTTCAGATGG
En15	ATTGAGACGGGATAAGCGCCA	AAGGTCTACAGGCCAACCCT
GAPDH	AAGGGCTCATGACCACAGTC	TGTGAGGGAGATGCTCAGTG
**Primers used for ChIP-qPCR**		
**Regions**	**Forward primer (5′-3′)**	**Revers primer (5′-3′)**
En8-1	AGCACTCACGACTTCCCAAA	AAAGGCCACCGTGACCAATTA
En8-2	GCAGGCAGATTACACCCATGA	CCTTTTCGTGGATGTTGCCA
En8-3	AGGCTTTCTGTGGGTTCTCTG	GCAAGCATCTCACAGTGCAAA
En9-1	TAGCTGTTCCTTCCGGTGAC	TAAGCGCACTGTCTTGCCTC
En9-2	CAGCACAGGATGGCAAAACA	AACTTGGCACCGAGGCTAAT
En9-3	ACCGCCAGCAATTTACAAGG	AAACAGGGCTGGACAGATCA
En10-1	AACCCACCTTCTGCCTAACC	TTTGAACACTCCAGTCCCAGG
En10-2	GGCCATTAGCACGCAGTACA	GCTTTTAGCCCTCAGGTTGC
En10-3	CAAGCTACCCCAGGTCAGAG	GAAAGATAGCCCCCACTGCC
En14-1	TCAAAAGGATGAAGGGGAGACA	CAGGCACTGTGCACTTAGTAG
En14-2	AAAACTTACATGCCACCGCT	ACTGAAAATGGCAACCACAGC
En14-3	TACCAGGGAACCGGATGAAA	TCGCCTAGTGGGAACTGTTA
En14-4	CCCCATCTGAAACTTGACGC	TTTAGAAGGCACTCAGCCAGA
En15-1	GGGTCTCCTGACTACTGCAAC	TCTGTGCCGTAGATGCCCAG
En15-2	CTTTCAGTGGTGTTGCACGG	GCGTGTGCAGTAGTACCTGT
En15-3	GCTTAGTTGCACGACAGTGG	TGTCCCCCTACACCAGAAGT
**Primers used for 3C-PCR with promoter**		
**Regions**	**Enhancer primer (5′-3′)**	**Promoter primer (5′-3′)**
En8-a	CCTCTTACCCACTGAGCCCCCATTACTC	GAGTTCCTTTGGTGTGTGTGACAGGTGAG
En8-b	ACACAAGACTGTCCACTCCCCATCTCTC	GAGTTCCTTTGGTGTGTGTGACAGGTGAG
En8-c	AGAGACACCTCAAACCTCACGTCACTGA	GAGTTCCTTTGGTGTGTGTGACAGGTGAG

En8-d	GCATTCAGGAGGGATGGCTGTTTCACTT	GAGTTCCTTTGGTGTGTGTGACAGGTGAG
En8-e	CCAACAAGGGACTGCATGAAAAAGACTTCC	GAGTTCCTTTGGTGTGTGTGACAGGTGAG
En8-f	TTCTCTTTTGTGTCGAGGGTGACCAGGAC	GAGTTCCTTTGGTGTGTGTGACAGGTGAG
En8-g	CATACACACAGCAGCCCTTACCAAGTGAG	GAGTTCCTTTGGTGTGTGTGACAGGTGAG
En8-h	CTAATGGCTGAGCTGTCTCTCCAGACCT	GAGTTCCTTTGGTGTGTGTGACAGGTGAG
En8-i	CCCAGATGAATAAGACTGATGACCCCTCCC	GAGTTCCTTTGGTGTGTGTGACAGGTGAG
En8-j	GAGGAAGGAAAGAAAGACGCATCTCCGT	GAGTTCCTTTGGTGTGTGTGACAGGTGAG
En8-k	TGTCAGGGAACAGAAGCCAGAGGTTACC	GAGTTCCTTTGGTGTGTGTGACAGGTGAG
En14-a	GCCAGCGGTCTCTGTCATTTCTACAGTAT	GAGTTCCTTTGGTGTGTGTGACAGGTGAG
En14-b	ACGCCAGCTGACTTCTCTACACCTTCTTC	GAGTTCCTTTGGTGTGTGTGACAGGTGAG
En14-c	TTCCAGAAGTCCCGCCATTATCCTGCTCA	GAGTTCCTTTGGTGTGTGTGACAGGTGAG
En14-d	CTCTGAAAGCACAGTGGCAGTGACGTTTT	GAGTTCCTTTGGTGTGTGTGACAGGTGAG
En14-e	GGCAGAAGATCAGCCTGTAGTTCCTCATT	GAGTTCCTTTGGTGTGTGTGACAGGTGAG
En14-f	TTAAGACCCCCCCTGTTTCATCCTCCTGAG	GAGTTCCTTTGGTGTGTGTGACAGGTGAG
En14-g	CACGGCACAAAACCTACACCTACACACAG	GAGTTCCTTTGGTGTGTGTGACAGGTGAG
En14-h	CAAGTCTGTGACCCTCCCTCTCTGAAATTG	GAGTTCCTTTGGTGTGTGTGACAGGTGAG
En14-i	CTGGTGACCTGAGTCCGATATACAGAACC	GAGTTCCTTTGGTGTGTGTGACAGGTGAG
En14-j	GGGAGGGGGGCATATGGTTTCATAGAGAT	GAGTTCCTTTGGTGTGTGTGACAGGTGAG
En14-k	CCCACACAATCACCTCTCTAACCTTGCTT	GAGTTCCTTTGGTGTGTGTGACAGGTGAG
En14-l	GCAAGCAGCCAATCGTTTCTTTCGTTAAGC	GAGTTCCTTTGGTGTGTGTGACAGGTGAG
En14-m	AGATAAAGAACCAGCCCCACAGGACAAGGG	GAGTTCCTTTGGTGTGTGTGACAGGTGAG
ctrl	AGGGAAGGGGCTCAGTGGTTA	GTCTACCACCGTGGATGTGG
**Primers used for 3C-PCR with enhancers**		
**Regions**	**Enhancer primer (5′-3′)**	**Enhancer primer (5′-3′)**
En8-e-En8-a	CCAACAAGGGACTGCATGAAAAAGACTTCC	CCTCTTACCCACTGAGCCCCCATTACTC
En8-e-En8-b	CCAACAAGGGACTGCATGAAAAAGACTTCC	ACACAAGACTGTCCACTCCCCATCTCTC

En8-e-En8-c	CCAACAAGGGACTGCATGAAAAAGACTTCC	AGAGACACCTCAAACCTCACGTCACTGA
En8-e-En8-d	CCAACAAGGGACTGCATGAAAAAGACTTCC	GCATTCAGGAGGGATGGCTGTTTCACTT
En8-e-En8-f	CCAACAAGGGACTGCATGAAAAAGACTTCC	TTCTCTTTTGTGTCGAGGGTGACCAGGAC
En8-e-En8-g	CCAACAAGGGACTGCATGAAAAAGACTTCC	CATACACACAGCAGCCCTTACCAAGTGAG
En8-e-En8-h	CCAACAAGGGACTGCATGAAAAAGACTTCC	CTAATGGCTGAGCTGTCTCTCCAGACCT
En8-e-En8-i	CCAACAAGGGACTGCATGAAAAAGACTTCC	CCCAGATGAATAAGACTGATGACCCCTCCC
En8-e-En8-j	CCAACAAGGGACTGCATGAAAAAGACTTCC	GAGGAAGGAAAGAAAGACGCATCTCCGT
En8-e-En8-k	CCAACAAGGGACTGCATGAAAAAGACTTCC	TGTCAGGGAACAGAAGCCAGAGGTTACC
En8-f-En8-a	TTCTCTTTTGTGTCGAGGGTGACCAGGAC	CCTCTTACCCACTGAGCCCCCATTACTC
En8-f-En8-b	TTCTCTTTTGTGTCGAGGGTGACCAGGAC	ACACAAGACTGTCCACTCCCCATCTCTC
En8-f-En8-c	TTCTCTTTTGTGTCGAGGGTGACCAGGAC	AGAGACACCTCAAACCTCACGTCACTGA
En8-f-En8-d	TTCTCTTTTGTGTCGAGGGTGACCAGGAC	GCATTCAGGAGGGATGGCTGTTTCACTT
En8-f-En8-g	TTCTCTTTTGTGTCGAGGGTGACCAGGAC	CATACACACAGCAGCCCTTACCAAGTGAG
En8-f-En8-h	TTCTCTTTTGTGTCGAGGGTGACCAGGAC	CTAATGGCTGAGCTGTCTCTCCAGACCT
En8-f-En8-i	TTCTCTTTTGTGTCGAGGGTGACCAGGAC	CCCAGATGAATAAGACTGATGACCCCTCCC
En8-f-En8-j	TTCTCTTTTGTGTCGAGGGTGACCAGGAC	GAGGAAGGAAAGAAAGACGCATCTCCGT
En8-f-En8-k	TTCTCTTTTGTGTCGAGGGTGACCAGGAC	TGTCAGGGAACAGAAGCCAGAGGTTACC
En8-h-En8-a	CTAATGGCTGAGCTGTCTCTCCAGACCT	CCTCTTACCCACTGAGCCCCCATTACTC
En8-h-En8-b	CTAATGGCTGAGCTGTCTCTCCAGACCT	ACACAAGACTGTCCACTCCCCATCTCTC
En8-h-En8-c	CTAATGGCTGAGCTGTCTCTCCAGACCT	AGAGACACCTCAAACCTCACGTCACTGA
En8-h-En8-d	CTAATGGCTGAGCTGTCTCTCCAGACCT	GCATTCAGGAGGGATGGCTGTTTCACTT
En8-h-En8-e	CTAATGGCTGAGCTGTCTCTCCAGACCT	CCAACAAGGGACTGCATGAAAAAGACTTCC
En8-h-En8-f	CTAATGGCTGAGCTGTCTCTCCAGACCT	TTCTCTTTTGTGTCGAGGGTGACCAGGAC
En8-h-En8-g	CTAATGGCTGAGCTGTCTCTCCAGACCT	CATACACACAGCAGCCCTTACCAAGTGAG
En8-h-En8-i	CTAATGGCTGAGCTGTCTCTCCAGACCT	CTAATGGCTGAGCTGTCTCTCCAGACCT
En8-h-En8-j	CTAATGGCTGAGCTGTCTCTCCAGACCT	CCCAGATGAATAAGACTGATGACCCCTCCC
En8-h-En8-k	CTAATGGCTGAGCTGTCTCTCCAGACCT	GAGGAAGGAAAGAAAGACGCATCTCCGT

En8-e-En14-a	CCAACAAGGGACTGCATGAAAAAGACTTCC	GCCAGCGGTCTCTGTCATTTCTACAGTAT
En8-e-En14-b	CCAACAAGGGACTGCATGAAAAAGACTTCC	ACGCCAGCTGACTTCTCTACACCTTCTTC
En8-e-En14-c	CCAACAAGGGACTGCATGAAAAAGACTTCC	TTCCAGAAGTCCCGCCATTATCCTGCTCA
En8-e-En14-d	CCAACAAGGGACTGCATGAAAAAGACTTCC	CTCTGAAAGCACAGTGGCAGTGACGTTTT
En8-e-En14-e	CCAACAAGGGACTGCATGAAAAAGACTTCC	GGCAGAAGATCAGCCTGTAGTTCCTCATT
En8-e-En14-f	CCAACAAGGGACTGCATGAAAAAGACTTCC	TTAAGACCCCCCCTGTTTCATCCTCCTGAG
En8-e-En14-g	CCAACAAGGGACTGCATGAAAAAGACTTCC	CACGGCACAAAACCTACACCTACACACAG
En8-e-En14-h	CCAACAAGGGACTGCATGAAAAAGACTTCC	CAAGTCTGTGACCCTCCCTCTCTGAAATTG
En8-e-En14-i	CCAACAAGGGACTGCATGAAAAAGACTTCC	CTGGTGACCTGAGTCCGATATACAGAACC
En8-e-En14-j	CCAACAAGGGACTGCATGAAAAAGACTTCC	GGGAGGGGGGCATATGGTTTCATAGAGAT
En8-e-En14-k	CCAACAAGGGACTGCATGAAAAAGACTTCC	CCCACACAATCACCTCTCTAACCTTGCTT
En8-e-En14-l	CCAACAAGGGACTGCATGAAAAAGACTTCC	GCAAGCAGCCAATCGTTTCTTTCGTTAAGC
En8-e-En14-m	CCAACAAGGGACTGCATGAAAAAGACTTCC	AGATAAAGAACCAGCCCCACAGGACAAGGG
En8-f-En14-a	TTCTCTTTTGTGTCGAGGGTGACCAGGAC	GCCAGCGGTCTCTGTCATTTCTACAGTAT
En8-f-En14-b	TTCTCTTTTGTGTCGAGGGTGACCAGGAC	ACGCCAGCTGACTTCTCTACACCTTCTTC
En8-f-En14-c	TTCTCTTTTGTGTCGAGGGTGACCAGGAC	TTCCAGAAGTCCCGCCATTATCCTGCTCA
En8-f-En14-d	TTCTCTTTTGTGTCGAGGGTGACCAGGAC	CTCTGAAAGCACAGTGGCAGTGACGTTTT
En8-f-En14-e	TTCTCTTTTGTGTCGAGGGTGACCAGGAC	GGCAGAAGATCAGCCTGTAGTTCCTCATT
En8-f-En14-f	TTCTCTTTTGTGTCGAGGGTGACCAGGAC	TTAAGACCCCCCCTGTTTCATCCTCCTGAG
En8-f-En14-g	TTCTCTTTTGTGTCGAGGGTGACCAGGAC	CACGGCACAAAACCTACACCTACACACAG
En8-f-En14-h	TTCTCTTTTGTGTCGAGGGTGACCAGGAC	CAAGTCTGTGACCCTCCCTCTCTGAAATTG
En8-f-En14-i	TTCTCTTTTGTGTCGAGGGTGACCAGGAC	CTGGTGACCTGAGTCCGATATACAGAACC
En8-f-En14-j	TTCTCTTTTGTGTCGAGGGTGACCAGGAC	GGGAGGGGGGCATATGGTTTCATAGAGAT
En8-f-En14-k	TTCTCTTTTGTGTCGAGGGTGACCAGGAC	CCCACACAATCACCTCTCTAACCTTGCTT
En8-f-En14-l	TTCTCTTTTGTGTCGAGGGTGACCAGGAC	GCAAGCAGCCAATCGTTTCTTTCGTTAAGC

En8-f-En14-m	TTCTCTTTTGTGTCGAGGGTGACCAGGAC	AGATAAAGAACCAGCCCCACAGGACAAGGG
En8-h-En14-a	CTAATGGCTGAGCTGTCTCTCCAGACCT	GCCAGCGGTCTCTGTCATTTCTACAGTAT
En8-h-En14-b	CTAATGGCTGAGCTGTCTCTCCAGACCT	ACGCCAGCTGACTTCTCTACACCTTCTTC
En8-h-En14-c	CTAATGGCTGAGCTGTCTCTCCAGACCT	TTCCAGAAGTCCCGCCATTATCCTGCTCA
En8-h-En14-d	CTAATGGCTGAGCTGTCTCTCCAGACCT	CTCTGAAAGCACAGTGGCAGTGACGTTTT
En8-h-En14-e	CTAATGGCTGAGCTGTCTCTCCAGACCT	GGCAGAAGATCAGCCTGTAGTTCCTCATT
En8-h-En14-f	CTAATGGCTGAGCTGTCTCTCCAGACCT	TTAAGACCCCCCCTGTTTCATCCTCCTGAG
En8-h-En14-g	CTAATGGCTGAGCTGTCTCTCCAGACCT	CACGGCACAAAACCTACACCTACACACAG
En8-h-En14-h	CTAATGGCTGAGCTGTCTCTCCAGACCT	CAAGTCTGTGACCCTCCCTCTCTGAAATTG
En8-h-En14-i	CTAATGGCTGAGCTGTCTCTCCAGACCT	CTGGTGACCTGAGTCCGATATACAGAACC
En8-h-En14-j	CTAATGGCTGAGCTGTCTCTCCAGACCT	GGGAGGGGGGCATATGGTTTCATAGAGAT
En8-h-En14-k	CTAATGGCTGAGCTGTCTCTCCAGACCT	CCCACACAATCACCTCTCTAACCTTGCTT
En8-h-En14-l	CTAATGGCTGAGCTGTCTCTCCAGACCT	GCAAGCAGCCAATCGTTTCTTTCGTTAAGC
En8-h-En14-m	CTAATGGCTGAGCTGTCTCTCCAGACCT	AGATAAAGAACCAGCCCCACAGGACAAGGG
**Primers used for DNA cloning**		
**Regions**	**Forward primers (5′-3′)**	**Reverse primers (5′-3′)**
Wnt8a pro	CTAGCTAGCTAGTCACCTACAGTCGCCAAGAG	ATAACCGGTTTAGGTATAACACATGCCCGCAG
En8	TTCCATATGGAATTCCGCCCAGAACTGATGCGGTTA	CTAGCTAGCTAGAACCAATTCACTCCCTCGCT
En14	TTCCATATGGAATTCCAATGTCACCACAGTAGACCCT	CTAGCTAGCTAGGCGTCAAGTTTCAGATGGGG
Random sequences	TTCCATATGTCCTTGAGAGTTTTCGCCCC	CTAGCTAGCTCAGTGCTGCAATGATACCGC
